# A comparative study on patient safety culture among high-risk hospital staff in the context of the COVID-19 and non-COVID-19 pandemic: a cross-sectional study in Taiwan

**DOI:** 10.3389/fpubh.2023.1200764

**Published:** 2023-07-27

**Authors:** Chih-Hsuan Huang, Hsin-Hung Wu, Yii-Ching Lee

**Affiliations:** ^1^Business School, Hubei University of Economics, Wuhan, China; ^2^Hubei Enterprise Culture Research Center, Hubei University of Economics, Wuhan, China; ^3^Research Center of Hubei Logistics Development, Hubei University of Economics, Wuhan, China; ^4^Department of Business Administration, National Changhua University of Education, Changhua, Taiwan; ^5^Department of M-Commerce and Multimedia Applications, Asia University, Taichung, Taiwan; ^6^Faculty of Education, State University of Malang, Malang, East Java, Indonesia; ^7^Department of Health Business Administration, Hung Kuang University, Taichung, Taiwan

**Keywords:** COVID-19 pandemic, patient safety culture, safety attitudes questionnaire, cross-sectional study, high-risk hospital staff

## Abstract

The study aimed to compare the evolution of patient safety culture perceived by high-risk hospital staff in the context of the COVID-19 pandemic and non-COVID-19 pandemic and to examine the variations in patient safety culture across demographic variables. The study found that the COVID-19 pandemic has significantly impacted patient safety culture in healthcare settings, with an increased focus on safety climate, job satisfaction, teamwork climate, stress recognition, and emotional exhaustion. Safety culture and work stress vary among medical professionals of different age groups. To reduce stress, workload should be minimized, work efficiency improved, and physical and mental health promoted. Strengthening safety culture can reduce work-related stress, improve job satisfaction, and increase dedication towards work. The study recommends interventions such as psychological and social support, along with emotional management training, to reduce emotional exhaustion. Healthcare institutions can set up psychological counseling hotlines or support groups to help medical professionals reduce stress and emotional burden.

## 1. Introduction

Patient safety culture has gradually been attached importance by healthcare organizations around the world. Patient safety culture refers to employees’ shared beliefs, attitudes, values, norms, and behavioral characteristics regarding patient safety and is considered an important component of hospital culture that is oriented towards patient safety (1, 2). Studies indicated that healthcare institutions can have many positive outcomes if they are able to possess a good patient safety culture. Positive patient safety culture within a hospital benefits many patient-related outcomes, including increased satisfaction with care, reduced patient discomfort, decreased fall rates, and lower rates of procedural complications (3, 4). Research in the field of healthcare management has also confirmed the impact of patient safety culture on medical professionals. For instance, the decrease of turnover intention and personal burnout was related to the increase of patient safety culture perceived by medical staff (5). Other studies suggest that a favorable patient safety culture has been associated with positive outcomes such as improved teamwork and communication among hospital staff, greater job satisfaction, and enhanced well-being (6, 7).

During the COVID-19 pandemic, patient safety culture in high-risk units of hospitals has become particularly important. These high-risk units, such as intensive care units (ICU), emergency departments (ED), and operating rooms, are where medical personnel and patients are most vulnerable to infection. In general, medical professionals in high-risk units need to face various complex surgeries, treatments, and monitoring. If they lack proper preventive measures and protection awareness, it will increase the risk of COVID-19 infection (such as airborne and fluid transmission) and medical errors. Establishing a patient safety culture can improve medical professionals’ awareness of disease control and infection prevention, thereby improving the quality of medical services and further enhancing patient safety. However, few studies have explored the changes in patient safety culture among high-risk hospital staff that have occurred before and after the COVID-19 pandemic (1). The aim of this study was to examine the patient safety culture perceived by high-risk hospital staff in the context of the COVID-19 and non-COVID-19 pandemic. Specifically, we aimed to:

1. Compare how patient safety culture has evolved in the context of the COVID-19 pandemic and non-COVID-19 pandemic.
2. Examine the variations in patient safety culture across demographic variables in the context of the COVID-19 pandemic and non-COVID-19 pandemic.

## 2. Methods

### 2.1. Hospital setting

The research was conducted at a regional teaching hospital situated in Taichung City, which is ranked as one of the leading medical facilities in Taiwan. The hospital has a workforce of more than 2,000 employees and provides a diverse range of services, including 1,000 hospital beds and clinical education for medical professionals. It is composed of over 35 departments.

### 2.2. Data collection

In Taiwan, the Chinese version of the safety attitudes questionnaire (CSAQ) has been designated as the official questionnaire for evaluating patient safety culture since 2008. As per the current medical system in Taiwan, both the survey outcomes and the primary dataset need to be submitted electronically to the Joint Commission of Taiwan (JCT) via their website. Medical professionals (physicians and nurses) working in high-risk hospital environment (i.e., ICU and ED) in 2019 and 2020 were issued to compare the trends of patient safety culture in the period of COVID-19 and non-COVID-19 pandemic. The ICU at case hospital plays a pivotal role in managing critically ill patients, particularly during the COVID-19 pandemic. It encompasses comprehensive medical care, advanced life support, continuous monitoring, infection prevention and control, rigorous diagnostic and therapeutic interventions, and multidisciplinary collaboration, including departments of internal medicine, pediatrics, and respiratory medicine. Moreover, the ED case hospital also plays a critical role in managing urgent and emergent medical conditions, especially during the COVID-19 pandemic. It involves rapid triage, timely assessment and stabilization of patients, implementation of infection control measures, efficient diagnostic evaluation, and coordinated multidisciplinary care. The ICU had 72 medical professionals, while the ED had 41.

Medical professionals were asked to complete the questionnaire electronically through an email-delivered hyperlink. In the event that medical professionals lacked email addresses, a distinct account and password were supplied in hard copy format to enable access to the online questionnaire. Certain personnel opted for the paper-based format, whereby completed questionnaires were submitted to a dedicated box. All questionnaires were anonymized, devoid of any personally identifiable information.

### 2.3. Instruments

The study utilized the CSAQ to assess medical professionals’ perceptions on patient safety in high-risk hospital environment; the tool was translated from Dr. Sexton’s SAQ by the JCT in 2007 (8, 9). It originally consists of 30 items grouped into 6 dimensions. In 2014. the JCT added two new dimensions – emotional exhaustion (EE) and work-life balance (WLB) – in order to enhance the comprehensiveness of capturing the medical professionals’ perspectives on fatigue and work-life conditions (1, 6). The final questionnaire has 46 questions organized into eight categories: Teamwork Climate (TC), Safety Climate (SC), Job Satisfaction (JS), Stress Recognition (SR), Perceptions of Management (PM), Working Conditions (WC), Emotional Exhaustion (EE), and Work-Life Balance (WLB). Most of the dimensions are scored on a 5-point Likert-type scale to reflect level of agreement, ranging from 1 (“strongly disagree”) to 5 (“strongly agree”). In the questionnaire used in this study, a higher number of points in each dimension indicates a better result or a more positive outcome. This means that a higher score reflects a higher level of the dimension being assessed in each specific domain. Furthermore, EE was measured using reversed questions such that each respondent’s answer was adjusted. That is, the original answer of strongly agree represents the poorest perceptions of measuring outcome. Each item on the WLB questionnaire is rated on a 4-point Likert-scale almost never (less than 1 day per week), 4 points; sometimes (1–2 days per week), 3 points; most of the time (3–4 days per week), 2 points; and always (5–7 days per week), 1 point. The results of 2019 and 2020 demonstrate that the CSAQ dimensions have a high level of internal consistency and reliability, as evidenced by the Cronbach’s *α* values which ranged from 0.800 to 0.964.

### 2.4. Analysis methods

The data was managed and analyzed using SPSSv25. Descriptive statistics such as means, standard deviations, frequencies, and percentages were calculated. Bivariate analysis was conducted using one-way ANOVA and *t*-tests to examine the relationship between demographic and professional variables and the CSAQ dimensions. Additionally, Pearson’s coefficient (*r*) was used to identify any correlations between each of the CSAQ dimensions.

## 3. Results

### 3.1. The characteristics of samples

In both 2019 and 2020, a total of 113 questionnaires were distributed to the medical professionals in high-risk hospital environment. Out of these, 106 valid questionnaires were collected in 2019, resulting in a response rate of approximately 93.80%. In 2020, 102 valid questionnaires were collected, yielding a response rate of approximately 90.27%. To ensure consistency and comparability of the analysis results, this study employed a sample of 102 respondents for data analysis. The majority of participants in both 2019 and 2020 were female, accounting for 86.3% and 70.6% respectively, and most of them were nurses. Additionally, the majority of respondents were between the ages of 21 and 30, with 55.9% in 2019 and 66.7% in 2020 falling within this age range. The proportion of supervisors among respondents increased from 3.9% in 2019 to 8.8% in 2020. Furthermore, the results of both 2019 and 2020 indicated that most of the respondents had a college degree or above, with 95.1% and 96.1%, respectively, holding this level of education. In terms of work experience, nearly half of the respondents (about 47%) reported having worked for more than 5 years. Additionally, respondents in both years reported frequently needing to contact patients, with 91.1% in 2019 and 92.2% in 2020 indicating that this was a regular part of their job duties.

Overall, the majority of participants in both years were female, with nurses comprising the largest group. Additionally, a significant proportion of respondents fell within the 21–30 age range. The number of supervisors among the respondents increased from 2019 to 2020. Most of the participants held a college degree or above. Nearly half of the respondents reported having more than five years of work experience. Moreover, contacting patients was identified as a regular aspect of their job duties for the respondents in both years. These findings highlight the demographic characteristics, educational background, work experience, and job responsibilities of the healthcare professionals surveyed in this study.

### 3.2. Comparison of the CSAQ dimension in 2019 and 2020

The 2019 results of the statistical analysis demonstrated that TC (mean value is 3.72) has the highest mean value, whereas WLB (mean value is 2.72) has the lowest average value. The results of 2020 demonstrated that TC had the highest average value of 3.63, while WLB had the lowest mean value of 2.69. The Cronbach’s alpha values for the 2019 and 2020 CSAQ dimensions were ranged from 0.800 to 0.947. As shown in Table 1, most mean scores in 2020 were slightly lower than those in 2019, but these changes were not statistically significant (*p* > 0.05), except for the mean EE score. The mean EE score in 2020 was significantly higher than that in 2019 (*p* < 0.05).

**Table 1:** The CSAQ dimension in 2019 and 2020.

CSAQ dimension	2019			2020			*t*	*p-value*
	Max.	Mean (SD)	*ɑ*	Max.	Mean (SD)	*ɑ*		
TC	5.00	3.72 (0.83)	0.873	5.00	3.63 (0.67)	0.807	0.834	0.406
SC	5.00	3.62 (0.74)	0.900	5.00	3.58 (0.62)	0.815	0.452	0.652
JS	5.00	3.54 (0.91)	0.947	5.00	3.37 (0.94)	0.964	1.457	0.148
SR	5.00	3.48 (0.90)	0.881	5.00	3.52 (0.78)	0.827	−0.365	0.716
PM	5.00	3.49 (0.84)	0.865	5.00	3.36 (0.69)	0.800	1.199	0.233
WC	5.00	3.56 (0.77)	0.885	5.00	3.47 (0.72)	0.866	0.871	0.386
EE	5.00	2.98 (0.83)	0.935	5.00	3.14 (0.67)	0.876	−1.951	0.046*
WLB	4.00	2.72 (0.56)	0.858	4.00	2.69 (0.65)	0.881	0.416	0.679

*p < 0.05.

### 3.3. Comparison of correlations of the CSAQ dimensions in 2019 and 2020

Tables 2, 3 show the Pearson’s correlation coefficients between the dimensions of CSAQ for 2019 and 2020. The results for both years indicate a high degree of significant correlation between SC and TC. In 2019, the results also showed a high degree of significant correlation between JS and TC as well as SC. The PM was significantly correlated with TC, SC, and JS. Furthermore, the results for both 2019 and 2020 showed a high degree of significant correlation between WC and TC as well as the PM. Most dimensions of patient safety culture were found to be significantly negatively correlated with EE.

**Table 2:** Correlations matrix among CSAQ dimensions in 2019.

CSAQ Dimension	1	2	3	4	5	6	7	8
1.TC	1							
2.SC	0.905**	1						
3.JS	0.725**	0.740**	1					
4.SR	0.129	0.139	0.020	1				
5.PM	0.770**	0.800**	0.785**	0.111	1			
6.WC	0.726**	0.764**	0.717**	0.057	0.822**	1		
7.EE	−0.431**	−0.397**	−0.495**	0.337**	−0.457**	−0.360**	1	
8.WLB	0.323**	0.386**	0.461**	0.078	0.519**	0.442**	−0.536**	1

**p < 0.01.

**Table 3:** Correlations matrix among CSAQ dimensions in 2020.

CSAQ Dimension	1	2	3	4	5	6	7	8
1.TC	1							
2.SC	0.814**	1						
3.JS	0.605**	0.582**	1					
4.SR	0.070	0.197*	0.124	1				
5.PM	0.664**	0.679**	0.601**	0.057	1			
6.WC	0.700**	0.667**	0.571**	0.052	0.839**	1		
7.EE	−0.288**	−0.220*	−0.209*	0.506**	−0.221*	−0.283**	1	
8.WLB	0.264**	0.232*	0.224*	−0.106	0.301**	0.296**	−0.244*	1

*p < 0.05; **p < 0.01.

### 3.4. Comparison of bivariable analysis of demographic in 2019 and 2020

To understand the factors affecting the respondents’ perceptions of patient safety culture in 2019 and 2020, a bivariate analysis including demographic and the CSAQ dimensions was conducted (see Tables 4, 5). There was no significant difference in mean CSAQ scores across different age groups in 2019. The data from 2020 showed that there were significant differences in the mean SC and SR scores among different age groups. Specifically, the mean SC score for the 51–60 age group was significantly higher than that of the 31–40 age group, and the mean SR score for the 51–60 age group was significantly higher than that of the 41–50 age group.

**Table 4:** Bivariable analysis of demographic and CSAQ dimensions in 2019.

	TC (*n*) Mean (SD)	SC (*n*) Mean (SD)	JS (*n*) Mean (SD)	SR (*n*) Mean (SD)	PM (*n*) Mean (SD)	WC (*n*) Mean (SD)	EE (*n*) Mean (SD)	WLB (*n*) Mean (SD)
Age group								
21–30 years	(57) 22.15 (4.20)	(57) 25.14 (4.34)	(57) 17.28 (3.94)	(57) 14.54 (3.19)	(57) 14.08 (2.86)	(57) 14.47 (2.84)	(57) 26.38 (7.61)	(57) 19.17 (3.86)
31–40 years	(28) 22.60 (6.40)	(28) 25.46 (7.03)	(28) 17.82 (5.57)	(28) 13.64 (4.28)	(28) 13.46 (4.40)	(28) 13.35 (3.48)	(28) 27.57 (6.82)	(28) 18.82 (3.62)
41–50 years	(13) 22.46 (3.61)	(13) 25.53 (4.01)	(13) 18.69 (4.66)	(13) 13.23 (3.34)	(13) 13.92 (2.72)	(13) 14.69 (2.75)	(13) 28.15 (7.40)	(13) 19.23 (3.98)
51–60 years	(4) 22.32 (4.99)	(4) 27.50 (7.50)	(4) 20.00 (5.77)	(4) 9.75 (2.87)	(4) 15.75 (4.37)	(4) 15.75 (4.34)	(4) 34.75 (7.50)	(4) 19.75 (8.50)
*p-value*	0.984	0.856	0.555	0.520	0.619	0.281	0.173	0.965
*Post-hoc*								
Gender								
Male	(14) 24.78 (3.96)	(14) 27.71 (4.53)	(14) 20.57 (4.12)	(14) 15.71 (3.22)	(14) 16.00 (2.88)	(14) 16.00 (3.03)	(14) 26.28 (9.54)	(14) 19.64 (3.41)
Female	(88) 21.93 (5.04)	(88) 25.00 (5.27)	(88) 17.26 (4.50)	(88) 13.65 (3.61)	(88) 13.63 (3.35)	(88) 13.96 (3.02)	(88) 27.42 (7.18)	(88) 19.02 (4.07)
*p-value*	0.047*	0.072	0.011*	0.048*	0.014*	0.021*	0.602	0.591
Educational level								
High school or less	(5) 21.60 (7.40)	(5) 25.80 (5.54)	(5) 19.20 (6.18)	(5) 12.80 (4.43)	(5) 14.20 (3.53)	(5) 15.40 (3.78)	(5) 32.60 (7.66)	(5) 21.60 (2.88)
Diploma or Bachelor	(97) 22.36 (4.89)	(97) 25.35 (5.25)	(97) 17.63 (4.51)	(97) 14.00 (3.59)	(97) 13.94 (3.38)	(97) 14.18 (3.06)	(97) 26.98 (7.43)	(97) 18.97 (4.00)
*p-value*	0.742	0.853	0.460	0.473	0.872	0.394	0.104	0.153
Experience in organization								
1–11 months	(19) 23.78 (5.05)	(19) 27.05 (5.53)	(19) 20.26 (3.85)	(19) 13.89 (4.29)	(19) 15.21 (3.20)	(19) 15.84 (2.89)	(19) 29.16 (8.09)	(19) 21.21 (2.82)
1–2 years	(16) 22.18 (5.50)	(16) 24.68 (5.87)	(16) 17.43 (4.36)	(16) 14.37 (3.15)	(16) 14.06 (4.02)	(16) 14.12 (3.57)	(16) 27.31 (7.68)	(16) 19.56 (3.96)
3–4 years	(19) 20.78 (4.51)	(19) 23.89 (4.81)	(19) 15.73 (4.24)	(19) 14.57 (3.62)	(19) 12.84 (3.43)	(19) 13.42 (3.07)	(19) 24.73 (7.03)	(19) 17.94 (3.74)
5–10 years	(32) 21.93 (4.91)	(32) 24.96 (4.76)	(32) 16.87 (4.81)	(32) 13.50 (3.41)	(32) 13.62 (3.18)	(32) 13.81 (2.91)	(32) 27.78 (7.90)	(32) 18.65 (4.35)
11–20 year	(12) 22.83 (5.00)	(12) 26.08 (5.43)	(12) 18.91 (4.07)	(12) 14.00 (4.22)	(12) 13.75 (2.76)	(12) 13.67 (2.26)	(12) 26.50 (5.48)	(12) 18.33 (4.39)
Above 21 years	(4) 24.75 (5.73)	(4) 28.25 (6.29)	(4) 19.25 (5.56)	(4) 12.75 (2.98)	(4) 16.25 (3.40)	(4) 16.25 (3.41)	(4) 28.25 (9.42)	(4) 18.75 (3.77)
*p-value*	0.576	0.426	0.038*	0.947	0.271	0.154	0.620	0.234
*Post-hoc*			1–11 > 3–4					
Managerial position								
Yes	(4) 29.50 (0.57)	(4) 33.50 (1.39)	(4) 23.75 (1.50)	(4) 14.25 (4.50)	(4) 17.75 (2.50)	(4) 17.75 (1.50)	(4) 30.75 (6.23)	(4) 19.75 (3.77)
No	(98) 22.03 (4.87)	(98) 25.04 (5.07)	(98) 17.46 (4.49)	(98) 13.92 (3.61)	(98) 13.80 (3.32)	(98) 14.10 (3.05)	(98) 27.12 (7.54)	(98) 19.08 (4.00)
*p-value*	0.001**	0.001**	0.007**	0.896	0.021*	0.020*	0.346	0.744

*p < 0.05; **p < 0.01.

**Table 5:** Bivariable analysis of demographic and CSAQ dimensions in 2020.

	TC (*n*) Mean (SD)	SC (*n*) Mean (SD)	JS (*n*) Mean (SD)	SR (*n*) Mean (SD)	PM (*n*) Mean (SD)	WC (*n*) Mean (SD)	EE (*n*) Mean (SD)	WLB (*n*) Mean (SD)
Age group								
21–30 years	(68) 21.73 (3.92)	(68) 24.86 (3.99)	(68) 16.66 (4.86)	(68) 14.32 (3.25)	(68) 13.50 (2.85)	(68) 13.85 (2.79)	(68) 24.70 (6.00)	(68) 18.83 (4.76)
31–40 years	(21) 21.09 (4.25)	(21) 24.28 (4.59)	(21) 16.33 (4.70)	(21) 13.80 (2.69)	(21) 12.85 (2.53)	(21) 13.67 (3.10)	(21) 27.42 (5.82)	(21) 18.95 (4.32)
41–50 years	(10) 21.80 (3.99)	(10) 25.90 (5.27)	(10) 17.80 (3.55)	(10) 12.30 (2.71)	(10) 14.10 (2.80)	(10) 14.20 (2.93)	(10) 28.40 (5.10)	(10) 19.00 (3.94)
51–60 years	(3) 27.66 (3.21)	(3) 32.33 (3.78)	(3) 21.66 (2.88)	(3) 17.66 (2.08)	(3) 15.00 (2.64)	(3) 15.66 (4.50)	(3) 25.67 (10.78)	(3) 18.66 (6.80)
*p-value*	0.073	0.022*	0.275	< 0.050*	0.489	0.716	0.145	0.980
*Post-hoc*		51–60 > 31–40		51–60 > 41–50				
Gender								
Male	(30) 22.60 (3.65)	(30) 25.60 (4.44)	(30) 17.43 (4.98)	(30) 14.33 (3.03)	(30) 13.16 (2.71)	(30) 14.10 (2.85)	(30) 24.06 (5.91)	(30) 19.90 (5.15)
Female	(72) 21.44 (4.20)	(72) 24.84 (4.38)	(72) 16.61 (4.60)	(72) 14.02 (3.21)	(72) 13.59 (2.81)	(72) 13.81 (2.92)	(72) 26.33 (6.09)	(72) 18.44 (4.30)
*p-value*	0.193	0.433	0.425	0.658	0.478	0.658	0.087	0.146
Educational level								
High school or less	(2) 25.50 (4.94)	(2) 30.00 (5.65)	(2) 20.00 (3.38)	(2) 15.50 (0.70)	(2) 16.00 (1.97)	(2) 15.50 (0.70)	(2) 19.50 (2.12)	(2) 21.50 (0.70)
Diploma or Bachelor	(98) 21.72 (4.06)	(98) 24.97 (4.36)	(98) 16.77 (4.77)	(98) 14.08 (3.19)	(98) 13.45 (0.28)	(98) 13.91 (2.92)	(98) 25.82 (6.13)	(98) 18.93 (4.58)
Master or doctor degree	(2) 21.00 (4.24)	(2) 24.50 (4.94)	(2) 17.50 (3.53)	(2) 14.50 (3.53)	(2) 11.50 (0.71)	(2) 11.50 (0.70)	(2) 24.00 (6.10)	(2) 13.00 (4.59)
*p-value*	0.418	0.276	0.624	0.811	0.266	0.373	0.326	0.139
Experience in organization								
1–11 months	(8) 20.50 (4.14)	(8) 24.87 (4.45)	(8) 19.50 (2.87)	(8) 14.37 (1.50)	(8) 12.37 (2.19)	(8) 13.12 (1.95)	(8) 23.12 (4.18)	(8) 21.37 (4.06)
1–2 years	(25) 22.60 (3.82)	(25) 25.24 (4.24)	(25) 16.68 (4.88)	(25) 14.72 (3.25)	(25) 13.88 (3.04)	(25) 14.64 (3.13)	(25) 23.68 (5.53)	(25) 19.92 (4.98)
3–4 years	(21) 20.28 (3.84)	(21) 24.28 (3.87)	(21) 14.76 (5.64)	(21) 14.71 (3.24)	(21) 12.85 (2.78)	(21) 12.90 (2.54)	(21) 23.90 (6.06)	(21) 17.95 (4.57)
5–10 years	(29) 22.13 (4.04)	(29) 24.65 (4.19)	(29) 16.55 (3.81)	(29) 13.31 (3.27)	(29) 13.27 (2.68)	(29) 13.55 (2.62)	(29) 27.10 (6.07)	(29) 18.10 (4.22)
11–20 year	(14) 21.35 (4.37)	(14) 25.71 (5.67)	(14) 18.07 (4.25)	(14) 13.92 (3.12)	(14) 14.00 (2.82)	(14) 14.00 (2.88)	(14) 28.00 (4.50)	(14) 19.71 (4.22)
Above 21 years	(5) 25.20 (3.70)	(5) 28.40 (4.66)	(5) 20.60 (4.72)	(5) 13.40 (3.78)	(5) 15.40 (2.07)	(5) 17.40 (3.43)	(5) 32.20 (8.72)	(5) 15.60 (6.98)
*p-value*	0.125	0.536	0.049*	0.569	0.322	0.025*	0.007**	0.136
*Post-hoc*			21 > 3–4			21 > 3–4	21 > 1–2	
Managerial position								
Yes	(9) 24.33 (4.66)	(9) 29.22 (5.51)	(9) 21.66 (3.27)	(9) 15.44 (3.57)	(9) 15.66 (2.91)	(9) 16.33 (3.31)	(9) 29.66 (6.26)	(9) 18.88 (4.70)
No	(93) 21.53 (3.94)	(93) 24.66 (4.08)	(93) 16.38 (4.57)	(93) 13.98 (3.10)	(93) 13.25 (2.68)	(93) 13.66 (2.75)	(93) 25.27 (5.97)	(93) 18.87 (4.60)
*p-value*	0.048*	0.003**	0.001**	0.188	0.012*	0.008**	0.039*	0.911

*p < 0.05; **p < 0.01.

Based on the results of an independent sample t-test, it was found that the mean scores for the dimensions of the CSAQ in TC, JS, SR, PM, and WC were significantly higher for males compared to females in 2019. However, there was no significant effect of gender on any of the CSAQ dimensions in 2020.

There are differences in the perceptions of the patient safety culture among medical personnel with varying levels of work experience. The data from 2019 demonstrated that the mean JS score of medical personnel with 1–11 months of experience in the organization was significantly higher than that of those with 3–4 years of experience. In 2020, the mean JS and WC score for the above 21 years of experience in the organization was significantly higher than that of the 3–4 years, and the mean EE score for the above 21 years was significantly higher than that of the 1–2 years.

Medical personnel who hold managerial positions have a significantly higher mean score in TC, SC, JS, PM, and WC compared to those who do not hold managerial positions in both 2019 and 2020. According to the data from 2020, medical personnel who hold managerial positions have a significantly higher score in EE than those who do not hold such positions. Educational level and whether they have contact with patients at work did not affect the CSAQ dimension score.

## 4. Discussion

This study fills gaps in the existing literature by focusing specifically on healthcare professionals working in high-risk hospital environments such as intensive care units and emergency rooms, this study narrows down the scope of research to a specific subset of medical personnel. This targeted approach helps to provide more precise insights into the patient safety culture within these critical units. Furthermore, the study’s emphasis on physicians and nurses as the primary participants the COVID-19 and non-COVID-19 contexts adds to the existing literature by shedding light on the specific experiences and perceptions of this healthcare professional group, which is valuable in understanding the dynamics of patient safety culture during the pandemic.

It is worth noting that our results show that there may be some differences in patient safety culture between COVID-19 and non-COVID-19 pandemic. It is important to carefully interpret these results and consider factors that may affect patient safety culture, such as age, experience in organization, and managerial position. First, our research findings indicate that during the pandemic, both safety culture and work stress are crucial issues for medical professionals. There are significant differences in safety culture and stress perception among medical professionals of different age groups. The results of this research are consistent with the findings of Preti et al. (10), who reported significant variations in stress perception among healthcare workers of different age groups during the pandemic. We suggest that reducing the workload and improving work efficiency can significantly lower the work stress of medical professionals. This can be achieved by increasing staff numbers, using more efficient tools and technologies (such as electronic medical record systems, remote medical technologies, real-time monitoring and alert systems, etc.), and improving scheduling. Additionally, providing healthy diets and moderate exercise can help to reduce the physical and mental stress of medical professionals.

Next, strengthening the cultivation of safety culture can enhance the awareness of medical professionals and reduce work-related stress. The results of our research align with the findings of Chen et al. (11), who highlighted the importance of strengthening the safety culture in reducing work-related stress among medical professionals. They emphasized the need for support and active participation from hospital management in cultivating a positive safety culture through clear policies, training programs, rewards, and regular inspections. Thus, establishing and maintaining a positive safety culture requires the support and participation of hospital management. This can be achieved by developing clear policies and procedures, enhancing training and education (such as courses on preventing hospital infections, occupational health and safety awareness, etc.), rewarding and conducting regular inspections and evaluations (such as safety incident reporting analysis, safety indicators: hospital infection rate, medication error rate, fall rate, etc.).

Third, improving the job satisfaction of medical professionals during the pandemic is also a worthy issue of concern. Similarly, Rana et al. (12) found that career development opportunities and skill training significantly influenced job satisfaction among healthcare workers during the COVID-19 outbreak. We suggest that more career development opportunities and skill training should be provided for medical professionals, enabling them to gradually improve their professional level and skills, and thus increase job satisfaction. Additionally, raising the salary levels and providing more stable career development opportunities for medical professionals can increase their dedication and sense of recognition towards their work.

Fourth, this study demonstrates that medical professionals in managerial positions perform better in terms of patient safety culture than those in non-managerial positions. We recommend that it is important to clearly authorize medical professionals with their responsibilities and accountabilities in patient safety, ensuring that they understand their role and responsibility in patient safety. This includes ensuring that they know how to detect and report adverse events, identify and manage potential risk factors, and promote and implement best practices in patient safety. Encouraging medical professionals in managerial positions to participate in patient safety work and providing rewards and incentives for outstanding employees can be beneficial. This may include recognizing excellent employees, providing training and promotion opportunities, etc. This can help motivate medical professionals to invest more time and energy into improving patient safety.

Finally, research data from 2019 and 2020 also shows a significant negative correlation between EE and most dimensions of the CSAQ, except for stress cognition. EE represents the phenomenon where individuals gradually lose their ability to express positive emotions and experience fatigue, weariness, and negative emotional states such as depression, as a result of prolonged work pressure, psychological burden, and emotional communication (13). EE among medical professionals increases with longer working hours. This may be due to the prolonged work pressure and emotional communication during the pandemic. Interventions are needed at both the organizational and individual levels to reduce medical professionals’ EE. Similarly, Chen et al. (14) concluded that prolonged work pressure, psychological burden, and emotional communication contribute to the development of EE among healthcare workers. We suggest that psychological support can alleviate medical professionals’ emotional stress. Healthcare institutions can set up psychological counseling hotlines or dedicated support groups to help medical professionals reduce stress and emotional burden. Similarly, social support can also alleviate emotional burden. Medical professionals can establish connections with family and friends to seek emotional support. They can also participate in emotional management training to improve their emotional management skills.

## 5. Conclusion

In the context of the COVID-19 pandemic, there have been significant changes in patient safety culture. The pandemic has brought new challenges and risks to healthcare settings, leading to increased emphasis on infection control, communication, and teamwork. Healthcare organizations have had to rapidly adapt to the constantly changing environment, which has impacted patient safety culture.

Emotional issues of medical professionals during the pandemic are often overlooked or deemed unimportant. It is worth noting that the effects of emotional conditions on medical professionals can be long-lasting and can have a significant impact on both their physical and mental health. These issues can also interfere with their job performance, potentially compromising patient safety. Within demographic structures, patient safety culture may vary between the COVID-19 and non-COVID-19 contexts. Factors such as the age, years of experience, managerial position and may also impact changes in patient safety culture in response to the pandemic.

A number of recommendations for future research are given. Further studies in different hospital settings and countries are suggested to confirm the findings of this study and determine if similar changes in patient safety culture have occurred in other healthcare systems. Conducting longitudinal studies to track the changes in patient safety culture over time during pandemics. This will help in evaluating the effectiveness of interventions aimed at improving patient safety culture and identifying areas for further improvement. Moreover, qualitative research could be conducted to gain a deeper understanding of the experiences of healthcare staff during pandemics and how they perceive patient safety culture. This will help in identifying the barriers and facilitators to improving patient safety culture in pandemics.

## Data availability statement

The raw data supporting the conclusions of this article will be made available by the authors, without undue reservation.

## Ethics statement

This Study received ethical clearance from the Institutional Review Board of Cheng Ching General Hospital in Taichung City, Taiwan with approval number HP190028. The participation in the study implies consent for the use of the data.

## Author contributions

H-CH and W-HH conceived and designed the study and draw the manuscript. L-YC collected data and H-CH and L-YC analyzed the data. All authors contributed to the article and approved the submitted version.

## Funding

This work was supported by Hubei Provincial Department of Education with the grant number of B2022137, Hubei Enterprise Culture Research Center, and Research Center of Hubei Logistics Development.

## Conflict of interest

The authors declare that the research was conducted in the absence of any commercial or financial relationships that could be construed as a potential conflict of interest.

## Publisher’s note

All claims expressed in this article are solely those of the authors and do not necessarily represent those of their affiliated organizations, or those of the publisher, the editors and the reviewers. Any product that may be evaluated in this article, or claim that may be made by its manufacturer, is not guaranteed or endorsed by the publisher.
